# Modular affinity-labeling of the cytosine demethylation base elements in DNA

**DOI:** 10.1038/s41598-020-76544-x

**Published:** 2020-11-20

**Authors:** Fanny Wang, Osama K. Zahid, Uday Ghanty, Rahul M. Kohli, Adam R. Hall

**Affiliations:** 1 Virginia Tech-Wake Forest University School of Biomedical Engineering and Sciences, Wake Forest School of Medicine, Winston-Salem, NC 27101 USA; 2 Department of Medicine and Department of Biochemistry and Biophysics, Perelman School of Medicine, University of Pennsylvania, Philadelphia, PA 19104 USA; 3 Comprehensive Cancer Center, Wake Forest School of Medicine, Winston-Salem, NC 27101 USA; 4 Present Address: Wake Forest Innovations, Wake Forest School of Medicine, Winston-Salem, NC 27101 USA

**Keywords:** Methylation analysis, DNA, DNA repair enzymes, DNA methylation

## Abstract

5-methylcytosine is the most studied DNA epigenetic modification, having been linked to diverse biological processes and disease states. The elucidation of cytosine demethylation has drawn added attention the three additional intermediate modifications involved in that pathway—5-hydroxymethylcytosine, 5-formylcytosine, and 5-carboxylcytosine—each of which may have distinct biological roles. Here, we extend a modular method for labeling base modifications in DNA to recognize all four bases involved in demethylation. We demonstrate both differential insertion of a single affinity tag (biotin) at the precise position of target elements and subsequent repair of the nicked phosphate backbone that remains following the procedure. The approach enables affinity isolation and downstream analyses without inducing widespread damage to the DNA.

## Introduction

Composed structurally of a cytosine nucleobase with a methyl group at the fifth carbon atom, the epigenetic modification 5-methylcytosine (5mC) has an overall prevalence of ~ 4% (5mC/C) in the human genome^1^. It is the most widely studied DNA base variant, largely because of the early advent of a technique with which it could be probed; it was demonstrated^2,3^ as early as 1970 that exposure to sodium bisulfite is capable of deaminating cytosines and converting them to uracils, but that this chemical reaction is blocked by methylation. In combination with the growing availability of sequencing technologies, this simple treatment has enabled a large number of studies that have been able to determine the genomic positions of 5mC as well as highlight its importance in diverse biological processes. For example, physiologically, 5mC has been shown to occur primarily in symmetric CpG dinucleotides in mammalian genomes^4^, where it plays an important role in the regulation of gene expression^5^ and has consequently been implicated in a variety of diseases^6^ including cancer^7^.

**Figure 1:**
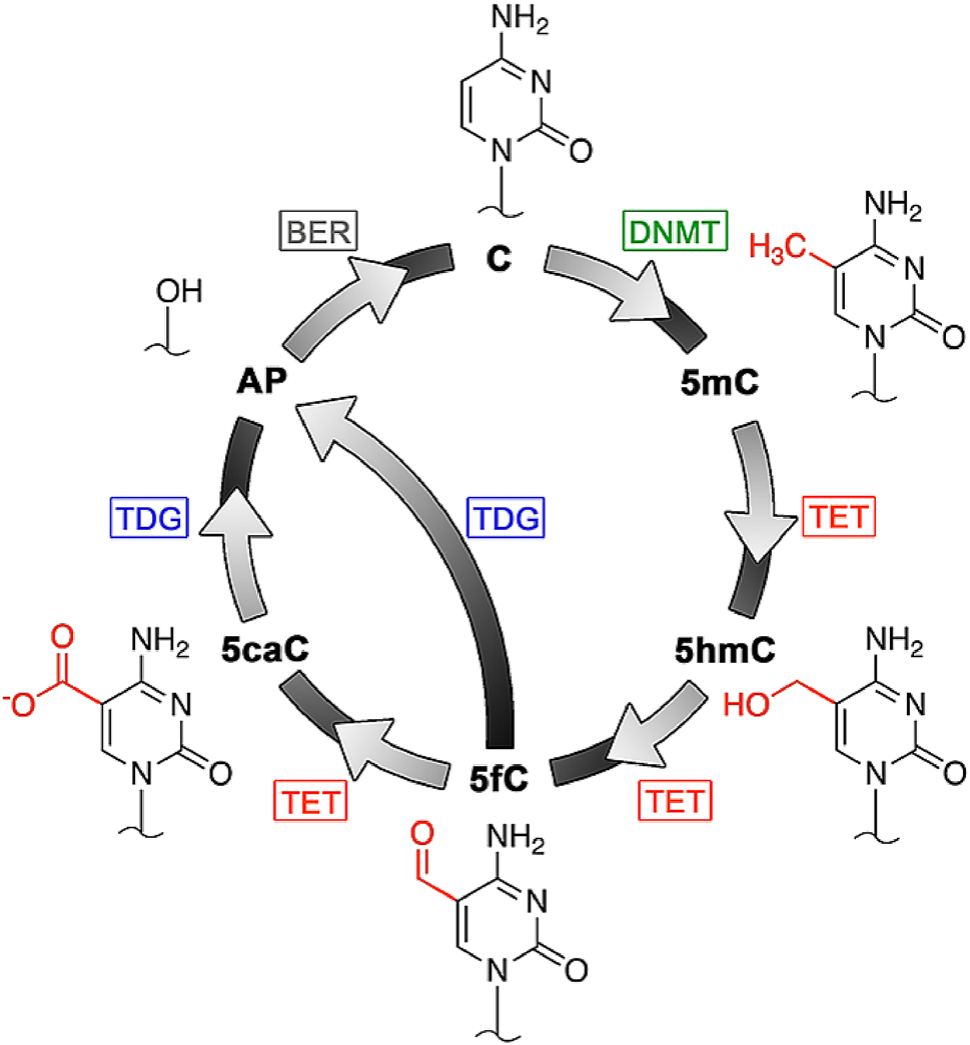
The cytosine methylation/demethylation pathway. Canonical cytosine (C) is methylated by a DNA methyltransferase (DNMT) to 5mC, which can then undergo TET oxidation to 5hmC, 5fC, and 5caC sequentially. Both 5fC and 5caC are recognized and excised by TDG, leaving an abasic (AP) site and enabling the BER pathway to install a canonical C.

While bisulfite treatment is the gold standard for DNA epigenetic analysis, it has two significant drawbacks. First, the procedure induces widespread damage to DNA in general. Bisulfite conversion of cytosines requires a single-strand target, so the process is typically carried out at elevated temperature. This, combined with the chemical reactivity of sodium bisulfite itself, results in substantial fragmentation of the DNA^8^ that can reduce its viability for downstream analyses and places practical limitations on the minimum starting DNA mass. Second, bisulfite conversion is limited in its intrinsic ability to resolve multiple cytosine modifications. The recent identification of the ten-eleven translocation (TET) family of enzymes^9,10^ has elucidated the pathway by which cytosine demethylation is achieved physiologically (Fig. 1): 5mC is oxidized in a stepwise fashion by TET to each of the three additional modified bases 5-hydroxymethylcytosine (5hmC), 5-formylcytosine (5fC), and 5-carboxylcytosine (5caC), the final two of which can be excised by thymine DNA glycosylase (TDG) and replaced with canonical cytosine upon completion of base excision repair (BER). Each of the three additional modified bases represents a potentially independent regulatory element, but bisulfite treatment has variable effects on them^11^, with 5mC and 5hmC each blocking conversion and 5fC and 5caC each able to be deaminated. Consequently, analyses incorporating conventional bisulfite treatment are inadequate to probe all components of the demethylation pathway. Innovative and effective strategies have been developed to expand possible base targets, but most still employ bisulfite^12–16^ (and thus still encounter the challenge of DNA damage above).

Driven partially by the interest generated by recent non-bisulfite approaches to the analysis of DNA epigenetics^17–22^, we employ a modular approach for installing a single affinity label at the precise locations of cytosine modifications and demonstrate adaptations to the process that enable all four elements of cytosine demethylation—5mC, 5hmC, 5fC, and 5caC—to be assessed. We show that modified bases can be replaced by a biotinylated nucleotide with high efficiency, providing a mechanism for selective isolation by e.g. streptavidin-driven affinity precipitation. In addition, we also show that the nicked backbone of the labeled DNA can be repaired via a ligation step to restore a structure that is viable for conventional genomic analyses like PCR and sequencing.

## Results

**Figure 2:**
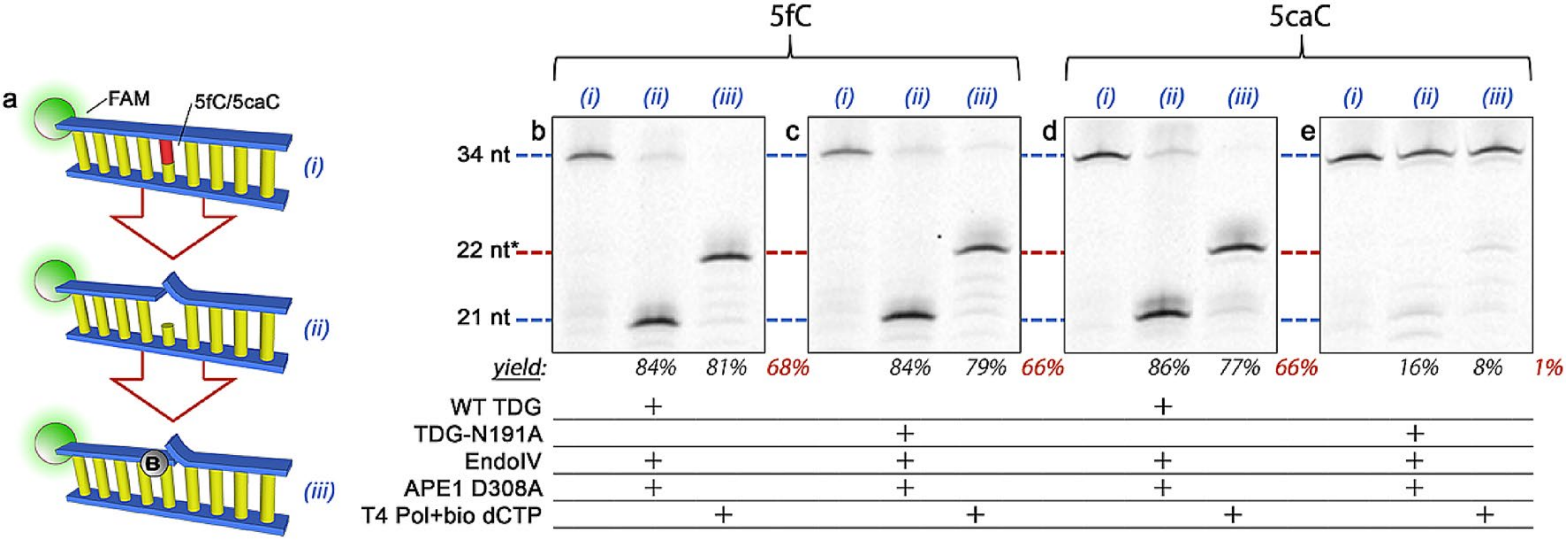
(**a**) Steps of the labeling scheme. DNA (i) containing a single modified base (red) is treated with a targeting glycosylase (a monofunctional glycosylase for illustration) to excise the base and an endonuclease to produce a site for polymerase activity (ii). A gap-filling polymerase is then used with a matched dNTP containing a biotin (‘B’) to install an affinity moiety at the precise location of the original modified base (iii). Illustration shows the fluorescent FAM label (green) employed for gel analyses of our constructs. Denaturing gel analyses of 34 nt DNA constructs featuring either 5fC (**b**–**c**) or 5caC (**d**–**e**) at base position 22 and labeled using either WT TDG (**b**, **d**) or TDG-N191A mutant (**c**, **e**). Lane (i): annealed oligonucleotide; lane (ii): following glycosylase/endonuclease treatment; lane (iii): following polymerase fill-in with a biotinylated nucleotide to yield a labeled product (red). Construct lengths at left apply to all gels and * indicates DNA length plus biotin tag. Directly below lanes (ii) and (iii) are listed target product yields from the previous step followed by the net yield in red. Full gel is shown in Supplementary Figure S1.

We recently^23^ reported on a general methodology for labeling single base modifications in DNA using elements of the BER pathway (Fig. 2a). Briefly, a glycosylase is used to specifically excise a target base from duplex DNA and leave an abasic (AP) site. Next, an AP endonuclease (EndoIV) is applied to cleave the phosphodiester backbone at the site, leaving a 3′ hydroxyl primed for polymerase incorporation. Finally, a gap-filling (i.e. non-displacing) polymerase and a biotinylated dNTP are used to replace the excised base, yielding an affinity tag located at the precise location of the target base modification. In previous work by us^23^ and others^24^, efficacy was demonstrated for a variety of bases that included uracil, 8-oxoguanine, and the methyladenine analog 1,N^6^-ethenoadenine. Here, we demonstrate a series of adaptations to enable recognition of all four elements of the cytosine demethylation pathway as well, including 5mC, 5hmC, 5fC, and 5caC.

First, we exploit the capability of wild-type (WT) TDG to excise both 5fC and 5caC. As a demonstration, we perform our full labeling procedure on model double-strand (ds) DNA oligonucleotides 34 bp in length that feature a single base modification positioned 22 nt from a fluorescent 5′ reporter (Fig. 2a; see *Materials and Methods*). Figure 2b–e shows the results of the sequential process for both of the modifications, as demonstrated by a denaturing gel that follows the single DNA strand featuring the 5′ fluorescent label. The initial 34 nt construct (lane 1) is first exposed to WT TDG glycosylase, along with AP endonuclease 1 (APE1 mutant D308A^25^ with reduced exonuclease activity) to displace the glycosylase^23^, which is known to bind tightly to the DNA substrate^26^. After a subsequent treatment with EndoIV to nick the DNA 5′ to the remnant AP site, we observe a shorter 21 nt product (lane 2), consistent with the position of the modification at base 22. After incubation with T4 DNA polymerase and biotinylated dCTP to fill the gap, the product increases in molecular weight to greater than 22 nt (lane 3); note that the shift appears larger than 1 nt because of the added mass and hydrophobicity of the attached biotin.

Our results show partial yields for each step (~ 80% or more) with WT TDG for both 5fC and 5caC, the constraints of which are primarily linked to substrate availability limited by incomplete annealing or enzyme saturation. These could be further improved through process optimization. The observed high efficiencies lead to net labeling yields of 68% and 66%, respectively, but also demonstrate a lack of differentiation with the procedure. To rectify this, we employ a mutant TDG (TDG-N191A) that has been shown^27^ to have selectivity for 5fC in particular. Repeating our procedure with this alternative glycosylase, we find that the 5fC construct yields the same characteristic shifts observed for WT TDG (Fig. 2d), indicating a comparable high net labeling yield (66%). In contrast, the 5caC construct results in minimal net yield (1%) through the same process (Fig. 2e), confirming the lack of 5caC recognition by the mutant TDG and indicating that no label is inserted. Consequently, the combined use of WT TDG and TDG-N191A in separate treatments can be used to deliver information about both modified bases through differential analysis. We speculate that another recently discovered^28^ mutant TDG (N157D) with specific recognition for 5caC only might also be used for completely independent analyses.

**Figure 3:**
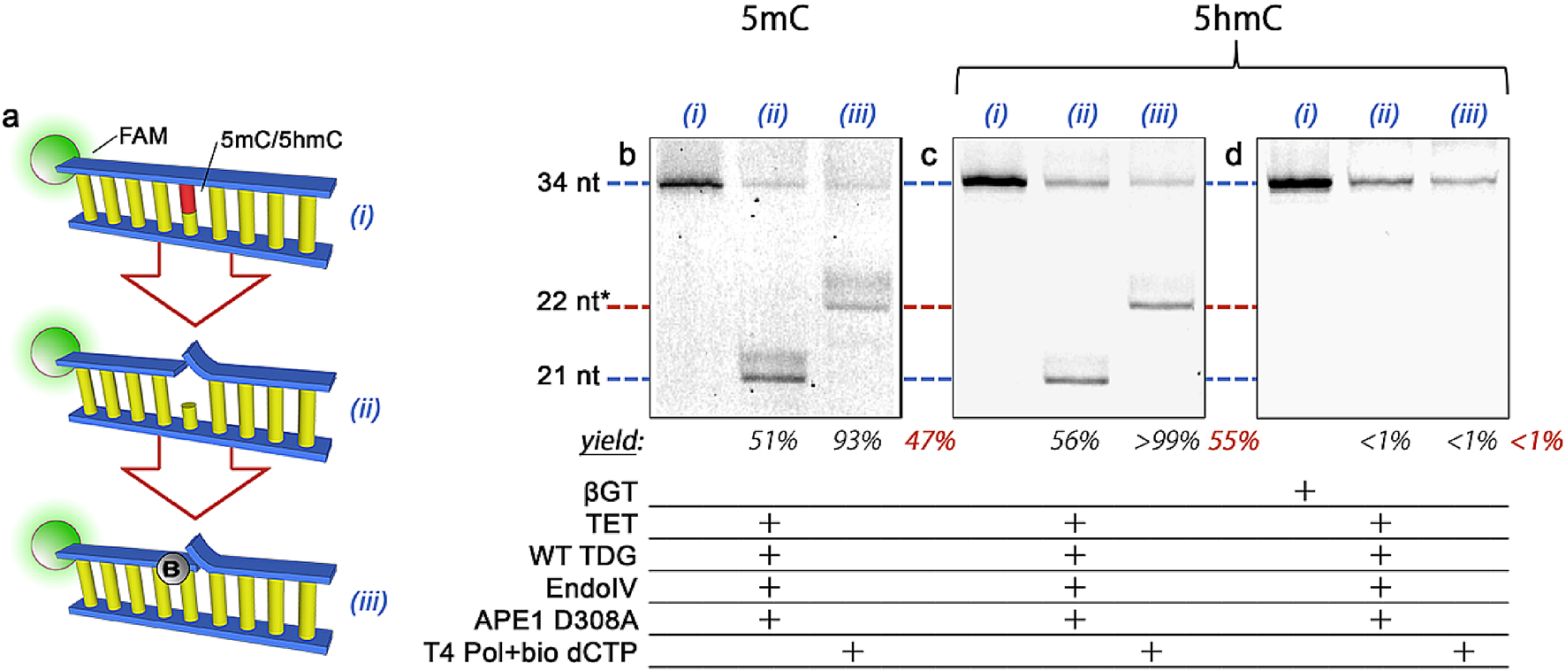
(**a**) Steps of the labeling scheme (see Fig. 2a for description). (**b**–**d**) Denaturing gel analyses of 34 nt DNA constructs featuring either 5mC (**b**) or 5hmC (**c**–**d**) at base position 22 and labeled using WT TDG following oxidation of each base with TET. In (**d**), a treatment with βGT prevents labeling of 5hmC specifically. Lane (i): annealed oligonucleotide ± βGT; lane (ii): following glycosylase/endonuclease treatment; lane (iii): following polymerase fill-in with a biotinylated nucleotide to yield a labeled product (red). Construct lengths at left apply to all gels and * indicates DNA length plus biotin tag. Directly below lanes (ii) and (iii) are listed target product yields from the previous step followed by the net yield in red. Full gels are shown in Supplementary Figure S3.

Having established protocols to assess 5fC and 5caC, we next investigate 5mC and 5hmC as base targets (Fig. 3a). For recognition of these two modifications collectively, we first employ TET to oxidize them and then subsequently carry out labeling with WT TDG as above. While TET oxidation converts these bases sequentially through each successive derivative, 5caC is the terminal product in the process. Consequently, the treatment can be performed to completion rather than requiring scheduled cessation to capture a particular base modification, in contrast to some existing applications of TET in demethylation analysis^29^. The results of this overall strategy using oligonucleotides with 5mC and 5hmC are shown in Fig. 3b,c, respectively. For both, an identical protocol results in effective insertion of biotinylated bases (47% and 55% net yield, respectively), demonstrating the effectiveness of WT TDG on the TET-oxidized substrates. The only difference between these substrates and the 5fC and 5caC ones above is the TET oxidation step and so we attribute the reductions in net yield to this process. From the values, we estimate TET efficiencies for converting 5mC and 5hmC to a recognizable base to be 69% and 81%, respectively. However, we also note that TET oxidation can be driven nearly to completion^30,31^ (Supplementary Fig. S2), suggesting that net yields equivalent to those achievable above are possible. No labeling was observed for either base without TET treatment, confirming that WT TDG has no intrinsic recognition for 5mC or 5hmC^32^.

As with 5fC and 5caC above, this procedure labels two base modifications simultaneously and so additional steps must be taken to discriminate 5mC and 5hmC. To achieve differentiation, we incorporate a treatment with β-glucosyltransferase (βGT), an enzyme that affixes a glucose moiety to 5hmC bases selectively. The presence of this bulky sugar disrupts the target recognition of TET and inhibits oxidation of 5hmC, thus preventing labeling with WT TDG. The effectiveness of this strategy is demonstrated in Fig. 3d, showing that βGT-treated 5hmC DNA yields no measurable product (< 1%) with the same treatment as above. In this way, the combination of TET with and without βGT in independent treatments enables analysis of both 5mC and 5hmC.

**Figure 4:**
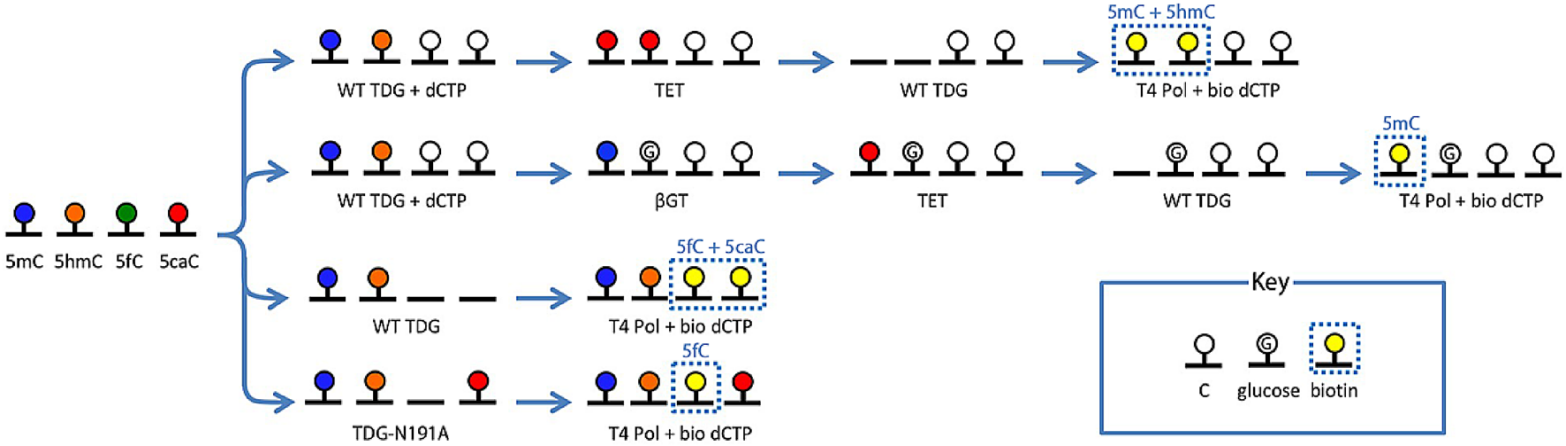
Labeling scheme for differentiating the four bases of the cytosine demethylation pathway with modular glycosylase labeling.

Because TET oxidizes 5mC, 5hmC, and 5fC bases in DNA to 5caC, the treatment renders all cytosine variants considered here susceptible to WT TDG recognition and labeling. This produces a potential complication in comprehensive analysis of all four demethylation elements independently. In practical terms, given the abundance of 5mC and 5hmC over 5fC and 5caC, the protocols are likely to be used for different profiling goals. A simple differential comparison between protocols with and without TET could be used to assign labeled DNA to either the 5mC/5hmC grouping or the 5fC/5caC grouping before further analysis. However, a more precise assessment could also be achieved by incorporating into the 5mC and 5hmC protocols an additional pretreatment with WT TDG in which canonical dCTP is incorporated rather than biotinylated nucleotides. This would preclude labeling of 5fC and 5caC selectively in subsequent steps and ultimately enable assessment of all four cytosine demethylation pathway base elements (Fig. 4).

**Figure 5:**
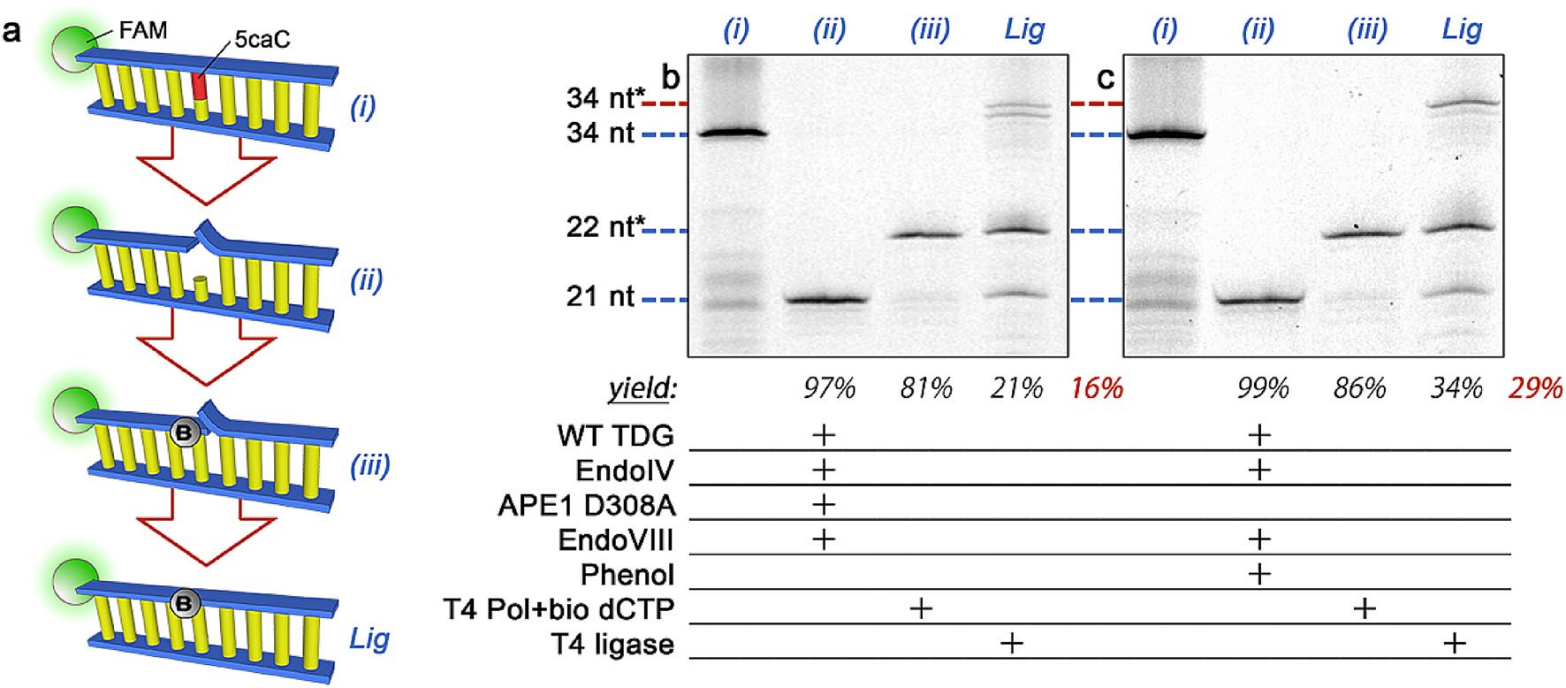
(**a**) Steps of the labeling scheme. (i)–(iii) the same as in Figs. 2 and 3. “Lig” indicates nick ligation. (**b**–**c**) Denaturing gel analyses of 34 nt DNA constructs featuring a single 5caC at base position 22. In each, the base is excised with WT TDG and the construct is treated with EndoIV to prepare the 3′ end of the gap, EndoVIII to remove the phosphate flap, T4 polymerase and biotinylated dCTP to label, and T4 ligase to repair the remaining nick. In (**b**), tightly-bound WT TDG is removed using APE1 D308A and in (**c**) it is removed by phenol exposure. Lane (i): annealed oligonucleotide; lane (ii): following glycosylase, APE1/phenol treatment, and endonuclease; lane (iii): following polymerase fill-in with a biotinylated nucleotide; lane labeled “Lig” is post ligation yielding a biotin-labeled construct with a repaired backbone (red). Construct lengths at left apply to both gels and * indicates DNA length plus biotin tag. Directly below lanes (ii), (iii), and “Lig” are listed target product yields from the previous step followed by the net yield in red. Full gels are shown in Supplementary Figure S6.

Our labeling strategy as we have demonstrated it thus far leaves a nick in the DNA backbone (c.f. Fig. 2a). This defect has no apparent negative effect on many applications including immunoisolation and single-molecule detection and quantification by solid-state nanopore^23^, but would be disruptive to other important analytical techniques like quantitative PCR or sequencing. Therefore, we next demonstrate a ligation step to repair the nick and restore the DNA structure (Fig. 5a).

There are two families of glycosylase: bifunctional and monofunctional^33^. Bifunctional glycosylases (i.e. those having AP lyase activity, like formamidopyrimidine-DNA glycosylase^34^) leave the labeled DNA strand primed for phosphate ester linkage and as a result give a substantial yield of repaired construct after direct ligation (48%, Supplementary Fig. S4). However, monofunctional glycosylases like TDG result in a phosphate flap that renders the nick a poor substrate for ligation. In principle, inclusion of an additional enzyme with independent AP lyase activity could remove the flap and enable subsequent ligation. Indeed, using another monofunctional glycosylase (uracil DNA glycosylase, or UDG) and a DNA construct featuring its recognized base, we find that incorporating the AP lyase endonuclease EndoVIII does enable the nick to be ligated with good yield (40%, Supplementary Fig. S5). We note for both of the above examples that the thermal stabilities of the short DNA strands remaining after the nick may limit the overall yields and that these may improve with longer constructs or genomic DNA fragments.

Critically, TDG in particular has the characteristic of maintaining strong binding affinity to the AP site after base excision^35^; this factor has necessitated^23^ the use of an active displacement element in our protocol in the form of AP endonuclease 1 (APE1). Unfortunately, either the specific activity of TDG binding to the DNA or its forcible removal appears to induce damage to the proximal substrate because we find a very low net yield (16%) of ligated construct and observe additional bands when employing the same protocol as for UDG (Fig. 5b). Increasing the EndoVIII concentration by up to 50% does not improve this yield (Supplementary Fig. S6). We note that while the precise nature of the damage is unclear, the observation that efficient base incorporation is achieved at the available 3′ end in the gap with T4 polymerase (c.f. Fig. 2) suggests that it is localized predominantly at the flap or at the base directly after the AP site. This could be related in part to the unusual binding conformation of TDG to DNA^36^.

To address this challenge, we finally investigate an alternative mechanism for TDG release intended to improve ligation yield by avoiding structural complications known to accompany APE1, including extensive DNA kinking^37^. For this, we use a phenol incubation following base excision by TDG. The low polarity of phenol makes it capable of inducing conformational changes in proteins exposed to the solvent^38^, driving hydrophilic residues into a more interior position while drawing hydrophobic residues to the surface in an inversion of the aqueous conformation. As such, we hypothesize that treatment of the TDG-bound DNA with phenol would result in release of the DNA with reduced substrate damage and sequestration of the TDG in the organic layer. To validate this, following TDG incubation, we introduce to the bound DNA a phenol solution at a final concentration of 25% (v/v). We then decant the aqueous layer to recover the released DNA, purify it via column purification, and continue labeling and ligation as with UDG. The results of this procedure demonstrate a significant improvement over the use of APE1 for TDG removal (Fig. 5c), achieving a net yield of ~ 29%. While this approach is not as effective as the protocol for glycosylases that do not demonstrate high binding affinity to AP sites, additional improvements may be instituted in the future to realize higher yields.

## Discussion

We report a method for affinity labeling the four components of the cytosine demethylation pathway in DNA, comprising 5mC, 5hmC, 5fC, and 5caC. While various methods exist for localizing individual modifications, a strength of our approach is that it builds on a modular labeling strategy^23^ for identification of diverse modified bases. This goal is achieved by employing the enzymatic constituents of the BER^39^ in which *(i)* a glycosylase is used to excise a target base, *(ii)* an endonuclease is used to hydroxylate the 3′ DNA end at the gap, and *(iii)* a polymerase is used to introduce a biotinylated base at the same position. Here, we exploit the recognition of TDG for some cytosine variants (5fC and 5caC) and enact a series of additional adaptations to the general protocol to permit the assessment of all four independent modifications: first, a TDG mutant (TDG-N191A) is employed to differentiate 5fC from 5caC; second, TET enzymes are used to oxidize 5mC and 5hmC and enable their joint recognition by WT TDG; and third, βGT is used to preferentially block 5hmC recognition and distinguish it from 5mC. Consequently, information about each variant can be attained by performing pairwise comparisons across the four closely related protocols. In addition, we also implement a ligation step to fully repair the DNA after labeling, resulting in undamaged duplex material.

The incorporation of biotin tags enables the enrichment and isolation of DNA fragments containing the modification or modifications of interest in a manner similar to immunoprecipitation^40,41^. Isolated products can subsequently be assessed by a broad range of analytical approaches including quantitative PCR or sequencing. In addition, the generalized method can also be applied easily to alternative labels like fluorophores or chemical linkers, provided that nucleotides synthesized to contain them are viable for polymerase incorporation. While modularity and diversity of base recognition are major advantages of our approach, another potential benefit is its directedness. In contrast to the widespread DNA damage induced by bisulfite exposure, the enzymatic activity employed is limited only to the base targets themselves. Thus, our methodology could enable improved analyses of small amounts of DNA, including those derived from inherently limited samples like liquid biopsies^42^, where target cell-free DNA (e.g. from a tumor) is often a very small population among a large background. Further, the modularity of our approach enables the investigation of a large suite of DNA modifications, including not only the cytosine demethylation elements demonstrated here but also bases like uracil, oxoguanine, and methyladenine^23^.

There are key challenges that remain with implementing our approach. For example, the overall labeling efficacy for each modification or set of modifications is reasonably high but must be improved. No part of the process is intrinsically limiting, so we anticipate this is possible through optimization of buffer conditions, enzyme concentration, temperature, and time. In addition, the repaired product yield following the ligation process is somewhat modest. This appears to be related to unidentified alteration to the phosphate backbone directly adjacent to the target modified base. With additional insight into the origins of this alteration, we expect that further protocol improvements will be possible. Due to the base excision step in our process, we also envision potential challenges with assessing symmetric modifications, i.e. modifications that are present on both strands of DNA. Critically, 5mC is often^4^ (though not always^43^) found in symmetric CpG dinucleotides in genomic DNA. It is unclear how TDG will act on symmetric modifications that have been oxidized by TET, however there is a theoretical risk of generated breaks on both strands of DNA. One potential solution could be to purposefully employ lower amounts of TET or TDG to limit excision efficiency, but another possibility could also include performing a single cycle of amplification prior to processing, thereby forming hemimethylated target sites that would not be prone to breakage.

In conclusion, we have described adaptations to an enzymatic procedure for affinity labeling that can be used to tag the four base modifications involved in cytosine demethylation. Overall, our approach adds to the epigenetics analytical toolbox by inducing low damage to DNA and providing modularity and extended target recognition, thereby progressing towards more comprehensive characterization of DNA modifications.

## Methods

### DNA constructs

Four sets of 34 nt-long DNA oligonucleotides featuring a fluorescent 5′ FAM label were purchased commercially (Integrated DNA Technologies, Coralville, IA) with the sequence 5′-CAG TTG AGG ATC CCC ATA ATG **C**GG CTG TTT TCT G-3′, in which the highlighted nucleotide (**C**) was replaced with 5mC, 5hmC, 5fC, or 5caC, respectively. While oligonucleotides were HPLC-purified, the combination of FAM-labeling and inclusion of modified bases could result in some off-target products manifesting as low mass banding on gel. This was particularly true for 5fC and 5caC (see Figs. 2, 5). Duplex constructs were formed by mixing 10 µM of each with its unmodified complementary sequence at a ratio of 1:1.2 in deionized water, incubating at 95 °C for 10 min, and gradually cooling to room temperature over two hours. Note that while robust products were confirmed by gel electrophoresis, the lack of salt in the annealing buffer could have reduced duplex availability somewhat, providing a potential source for reduced labelling yields.

### Protein expression

An APE1 mutant^25^ with reduced 3′–5′ exonuclease activity (D308A) was expressed using a method described previously^23^. The plasmid (generously provided by the Demple Lab at Stony Brook University) was transformed into BL21(DE3) cells and grown in 1 L LB broth at 37 °C until an OD600 of 0.6 was achieved, after which the cells were induced with 0.5 mM isopropyl β-d-thiogalactopyranoside (IPTG). An additional 90 min incubation was performed before harvesting cells by centrifugation, resuspending them in 50 mM HEPES–KOH (pH 7.5), 100 mM KCl, 1 mM EDTA, 0.1 mM dithiothreitol (DTT), and 10% (v/v) glycerol, and lysing them by two passages through an EmulsiFlex-C5 homogeniser (Avestin, Ottawa, Canada). Lysate was cleared by centrifuging for 20 min at 20,000 × *g* and the resulting mixture was loaded onto a 15 mL SP Sepharose column (GE Healthcare, Pittsburgh, PA). Elutions were performed with a linear gradient of KCl (100−750 mM) and then analyzed by SDS-PAGE to identify fractions containing the protein. These were pooled and dialyzed overnight at 4 °C against storage buffer containing 50 mM HEPES–KOH (pH 7.5), 200 mM KCl, 1 mM EDTA, 0.1 mM DTT, and 10% (v/v) glycerol and then concentrated using 10 kDa molecular weight cutoff centrifugal spin filter columns (EMD Millipore, Billerica, MA). The final protein concentration was determined analytically by Bradford protein assay (Bio-Rad, Hercules, CA) and aliquots were stored at − 20 °C prior to use.

For expression of WT TDG, we followed an existing protocol^30^ adapted from prior work^44^ with minor modifications. A plasmid for human TDG based on pET28 was transformed into BL21 (DE3) cells and grown in 1 L LB broth at 37 °C until the cultures reached an OD600 of 0.6. Then, they were gradually cooled to 16 °C, induced with 0.25 mM IPTG and incubated overnight. Harvesting was performed by centrifugation and retrieved cells were resuspended in 20 mL of TDG lysis buffer (50 mM sodium phosphate, pH 8.0, 300 mM NaCl, 25 mM imidazole) with protease inhibitors and then lysed by two passes through an EmulsiFlex-C5 homogeniser. The lysate was cleared by a 20 min centrifugation at 20,000×*g*, loaded onto a 1 mL column of HisPur cobalt resin (Fisher Scientific, Hampton, NH) equilibrated with TDG lysis buffer, and then bound by two applications of the lysate to the column under gravity flow. The column was washed with 20 mL of TDG lysis buffer and subsequently eluted by a linear gradient of imidazole (100–500 mM) into 1 mL aliquots that were then analyzed by SDS-PAGE. Fractions containing TDG were pooled and dialyzed overnight at 4 °C against TDG storage buffer (20 mM HEPES, pH 7.5, 100 mM NaCl, 1 mM DTT, 0.5 mM EDTA, 1% v/v glycerol). Dialyzed proteins were concentrated using 10 kDa molecular weight cutoff centrifugal spin filter columns. Final protein concentration was determined analytically by Bradford protein assay and aliquots were stored at − 80 °C prior to use.

A mutant TDG^27^ with no recognition for 5caC (TDG-N191A) was expressed in an identical fashion to WT TDG but using the mutant plasmid.

Human TET2-CS, the crystal structure variant of the enzyme (1129–1936 Δ1481–1843), was purified from insect cells as previously described^45^. Briefly, the construct, with an N-terminal FLAG tag, was subcloned into a pFastBac1 vector. After generation of baculovirus, 1 L of Sf9 cells were infected and cells were collected after 24 h and resuspended in lysis buffer (50 mM HEPES, pH 7.5, 300 mM NaCl, and 0.2% (v/v) NP-40) containing complete, EDTA-free Protease Inhibitor Cocktail (Roche, 1 tablet/10 mL). Cells were lysed by three passes through a microfluidizer at 15,000 psi and the lysate was cleared by centrifugation at 20,000×*g* for 30 min. The supernatant was then passed three times over a 1 mL packed column of anti-FLAG M2 affinity resin (Sigma). The column was washed three times with 10 mL of wash buffer (50 mM HEPES, pH 7.5, 150 mM NaCl, and 15% (v/v) glycerol). 1 column volume of elution buffer (wash buffer with 100 μg/mL 3 × FLAG peptide (Sigma) added) was then incubated on the column for 10 min followed by collection of the elution fraction. Serial elutions were similarly collected until no more protein was detected by the Bio-Rad Protein Assay. The three most concentrated fractions were pooled, aliquoted, and stored at − 80 °C.

### Gel electrophoresis

Denaturing gel electrophoresis was performed by first mixing 70 mL of a 23% gel matrix (22% acrylamide, 1% bis-acrylamide, 7 M urea in 1X tris/borate/EDTA (3:1:1) (TBE) buffer), 240 μL of 25% ammonium persulfate, and 42 μL tetramethylethylenediamine. After the mixture was cast, it was allowed to set for 30 min and then samples denatured at 95 °C for 10 min in formamide loading buffer (95% Formamide, 18 mM EDTA, and 0.025% each xylene cyanol and bromophenol blue) were loaded in 1X TBE (3:1:1) and run at 55 W for 120 min. Product yields were determined through quantification of band intensities by ImageJ analysis software^46^.

### Dual labeling 5fC and 5caC

40 pmol DNA was incubated with 3 μg wild-type TDG, 13.3 fg APE1 D308A, and 4 µg bovine serum albumin (BSA, New England Biolabs, Ipswitch, MA) in 20 µL HEMN.1 Buffer (200 mM HEPES, 1 M NaCl, 2 mM EDTA, 25 mM MgCl_2_) at 37 °C for 1 h to excise target bases and detach the TDG from the resulting AP site. After purifying the DNA with a Nucleotide Removal Kit (Qiagen, Valencia, CA), it was incubated with 20 U of Endonuclease IV (New England Biolabs), 100 U of Endonuclease VIII (New England Biolabs), and 4 µg BSA in 20 µL NEB2 buffer (50 mM NaCl, 10 mM tris–HCl, 10 mM MgCl_2_, 1 mM DTT, New England Biolabs) at 37 °C for 30 min to prime the gap for base incorporation. Then, 1.5 nmol of biotin-11-dCTP (C_28_H_44_N_7_O_16_P_3_S, Perkin Elmer, Waltham, MA) and 0.12 U of T4 polymerase having no exonuclease activity (Lucigen, Middleton, WI) were added and the mixture and incubated at 37 °C for an additional 30 min. The DNA was again purified with the Nucleotide Removal Kit and eluted in deionized water.

### Selective labeling of 5fC

An identical protocol was used as that described above for 5fC and 5caC, but substituting the TDG-N191A mutant for the WT TDG.

### Dual labeling 5mC and 5hmC

12.5 pmol DNA was incubated for 2 h at 37 °C with 1.5 µg of TET2-CS, 5 mM adenosine triphosphate (New England Biolabs), and 75 μM Fe(NH_4_)_2_(SO_4_)_2_ in 50 µL of reaction buffer containing 50 mM HEPES, 50 mM NaCl, 1 mM α-ketoglutarate, 2 mM l-ascorbic acid, and 1 mM DTT (pH 7.5) to fully oxidize both 5mC and 5hmC. The treated DNA was purified with the Nucleotide Removal Kit and eluted in deionized water. The above protocol for dual 5fC and 5caC was then followed for labeling.

### Selective labeling of 5mC

40 pmol DNA construct was incubated for 1 h with 10 pmol of UDP-Glucose (New England Biolabs) and 50 U of T4 phage βGT (New England Biolabs) in NEB4 buffer (50 mM potassium acetate, 20 mM tris–acetate, 10 mM magnesium acetate, 1 mM DTT, pH 7.9, New England Biolabs) at 37 °C. Then, the protected DNA was purified with the Nucleotide Removal Kit and eluted in deionized water. The above protocol for dual 5mC and 5hmC was then followed, resulting in labeling of 5mC alone.

### TDG release via phenol treatment

Where phenol was used to release TDG from the AP site, the protocol described above was employed with two exceptions. First, no APE1 was included in the base excision mixture (i.e. 40 pmol DNA, 3 μg WT TDG, and 4 µg BSA in HEMN.1 buffer). Second, directly following the excision step, an equal volume of phenol:chloroform:isoamyl alcohol (25:24:1) saturated with tris buffer (pH 8.0) was added and mixed by vortexing for 1 min, segregating the DNA construct into the aqueous (buffer) phase and the protein constituents (TDG, BSA) into the inorganic (phenol–chloroform) phase. The mixture was loaded into a phase-lock tube (5Prime, QuantaBio, Beverly, MA) and centrifuged at 14,000×*g* for 25 min and then an equal volume of pure chloroform was added and centrifuged at the same speed for an additional 20 min to remove any remnant phenol. Finally, the aqueous phase containing DNA was aspirated, purified with the Nucleotide Removal Kit, and eluted in deionized water. Subsequent protocol steps were then followed as described.

### Ligation

Labeled DNA with the phosphate flap removed (i.e. treated with Endonuclease VIII) was incubated with 400 U of T4 DNA Ligase (New England Biolabs) in T4 DNA Ligase buffer (50 mM tris–HCl 10 mM, MgCl_2_, 1 mM ATP, 10 mM DTT, pH 7.5) overnight at room temperature. The DNA was then purified with the Nucleotide Removal Kit and eluted in deionized water.

## Supplementary information

Supplementary Information.
