# Supplementary material for: Modular affinity-labeling of the cytosine demethylation base elements in DNA
Source: Sci Rep. 2020 Nov 20;10:20253. doi: 10.1038/s41598-020-76544-x (PMC7679407; doi:10.1038/s41598-020-76544-x)
Supplement: Supplementary file 1 — Supplementary Information. [file 41598_2020_76544_MOESM1_ESM.pdf]

Supplementary Information

Modular affinity-labeling of the cytosine demethylation base elements in DNA

Fanny Wang, Osama K. Zahid, Uday Ghanty, Rahul M. Kohli, and Adam R. Hall

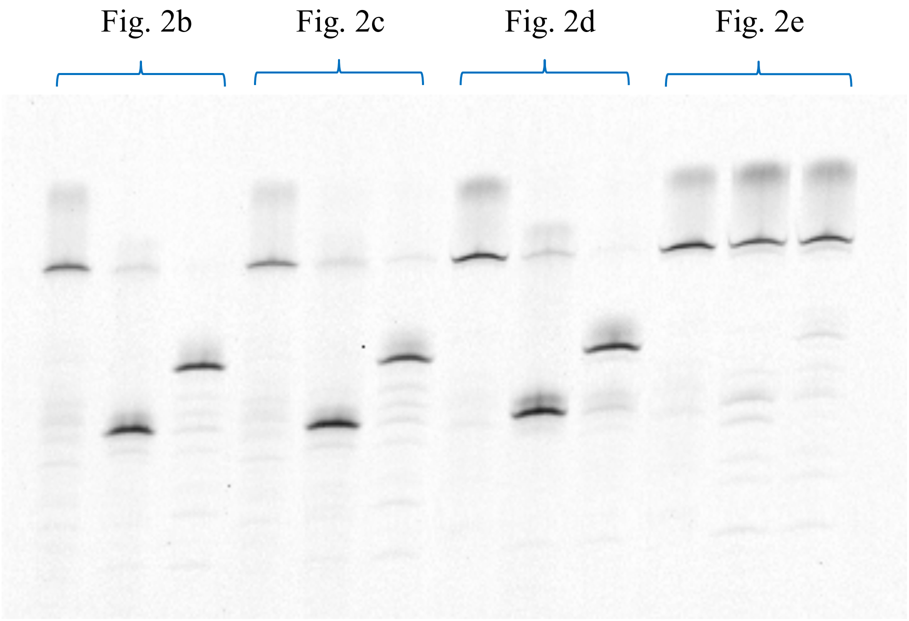

Supplementary Figure S1. *Full image of gels shown in Figure 2 of the main text, showing labeling of 5fC and 5caC*

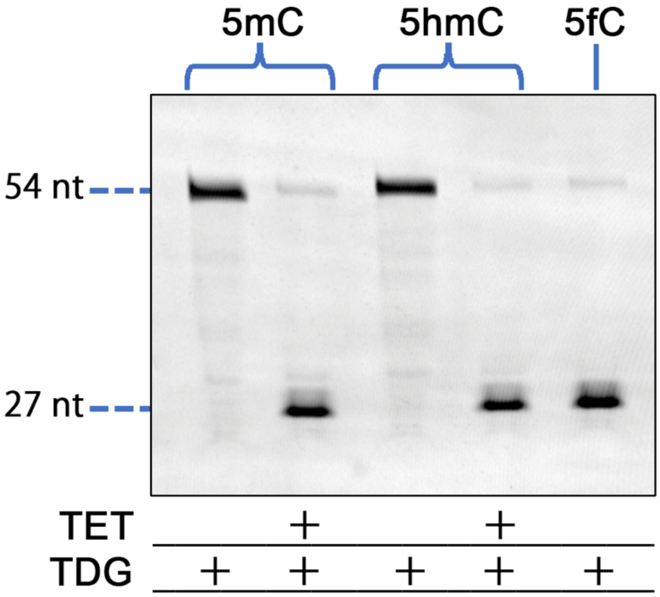

Supplementary Figure S2. *Denaturing gel analyses of 54 nt DNA constructs featuring a single modified base (5mC, 5hmC, or 5fC) at base position 27. Indicated constructs are treated with TET under optimized conditions to oxidize 5mC or 5hmC to 5fC/5caC and then all constructs are treated with WT thymine DNA glycosylase (TDG) to excise amenable bases. 5fC serves as a positive control that is intrinsically recognized by TDG, demonstrating 92% yield. Under these conditions, TET-treated 5mC and 5hmC constructs achieve yields of 88% and 89%, respectively, demonstrating nearly complete conversion of both modified bases. Construct descriptions and full protocol are provided in Supplementary Methods.*

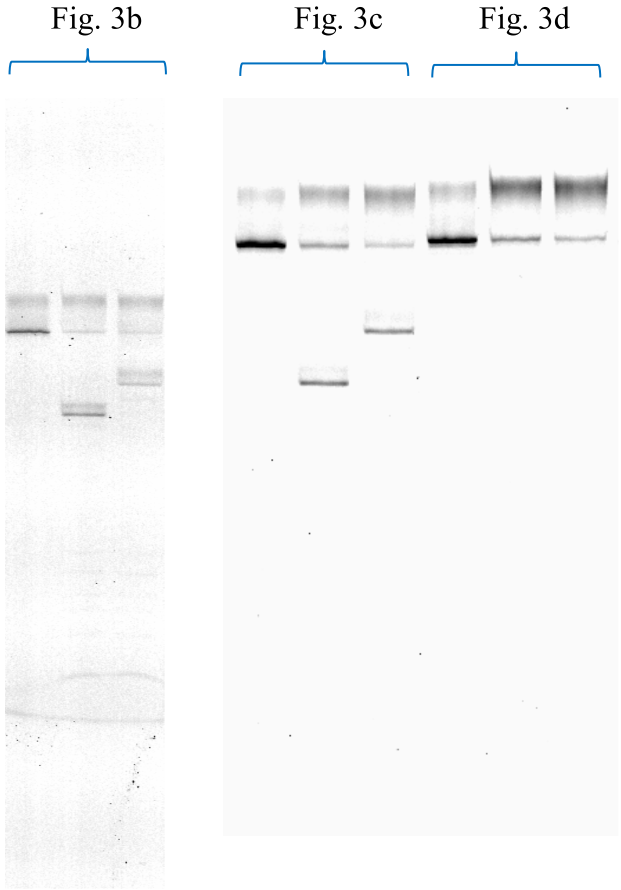

Supplementary Figure S3. *Full image of gels shown in Figure 3 of the main text, showing labeling of 5mC and 5hmC*

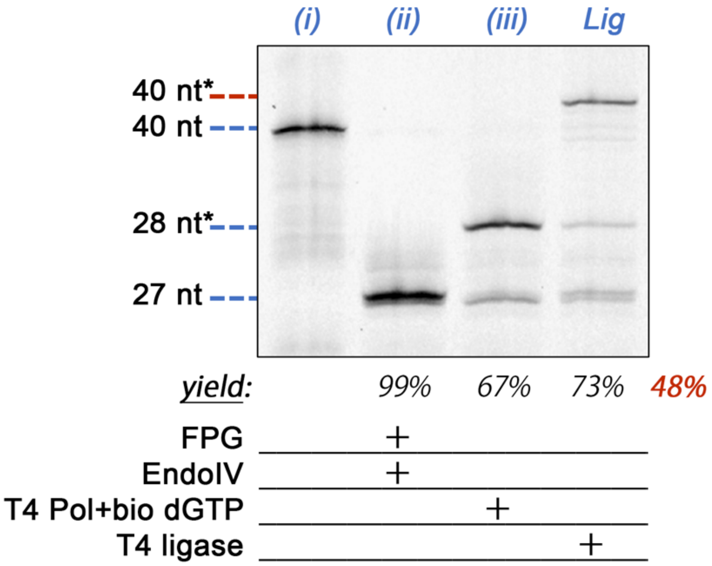

Supplementary Figure S4. *Denaturing gel analyses of 40 nt DNA constructs featuring a single 8-oxoguanine (oxoG) at base position 27. The base is excised with the bifunctional glycosylase formamidopyrimidine-DNA glycosylase (FPG) and the construct is treated with EndoIV to prepare the 3' end of the gap, T4 polymerase and biotinylated dGTP to label, and T4 ligase to repair the remaining nick. Lane (i): annealed oligonucleotide; lane (ii): following glycosylase/endonuclease treatment; lane (iii): following polymerase fill-in with a biotinylated nucleotide; lane "Lig" is post ligation yielding a biotin-labeled construct with a repaired backbone (red). Construct lengths at left apply to both gels and * indicates DNA length plus biotin tag. Directly below lanes (ii), (iii), and Lig are listed target product yields from the previous step followed by the net yield in red. Full gel shown in Supplementary Figure S7.*

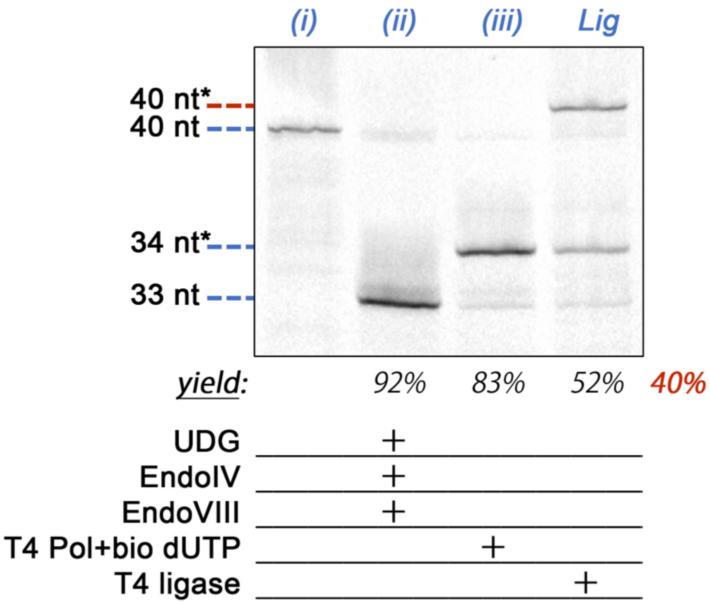

Supplementary Figure S5. *Denaturing gel analyses of 40 nt DNA constructs featuring a single uracil at base position 33. In each, the base is excised with the monofunctional glycosylase uracil DNA glycosylase (UDG) and the construct is treated with EndoIV to prepare the 3' end of the gap, EndoVIII to remove the phosphate flap, T4 polymerase and biotinylated dUTP to label, and T4 ligase to repair the remaining nick. Lane (i): annealed oligonucleotide; lane (ii): following glycosylase/endonuclease treatment; lane (iii): following polymerase fill-in with a biotinylated nucleotide; lane "Lig" is post ligation yielding a biotin-labeled construct with a repaired backbone (red). Construct lengths at left apply to both gels and * indicates DNA length plus biotin tag. Directly below lanes (ii), (iii), and Lig are listed target product yields from the previous step followed by the net yield in red. Full gel shown in Supplementary Figure S7.*

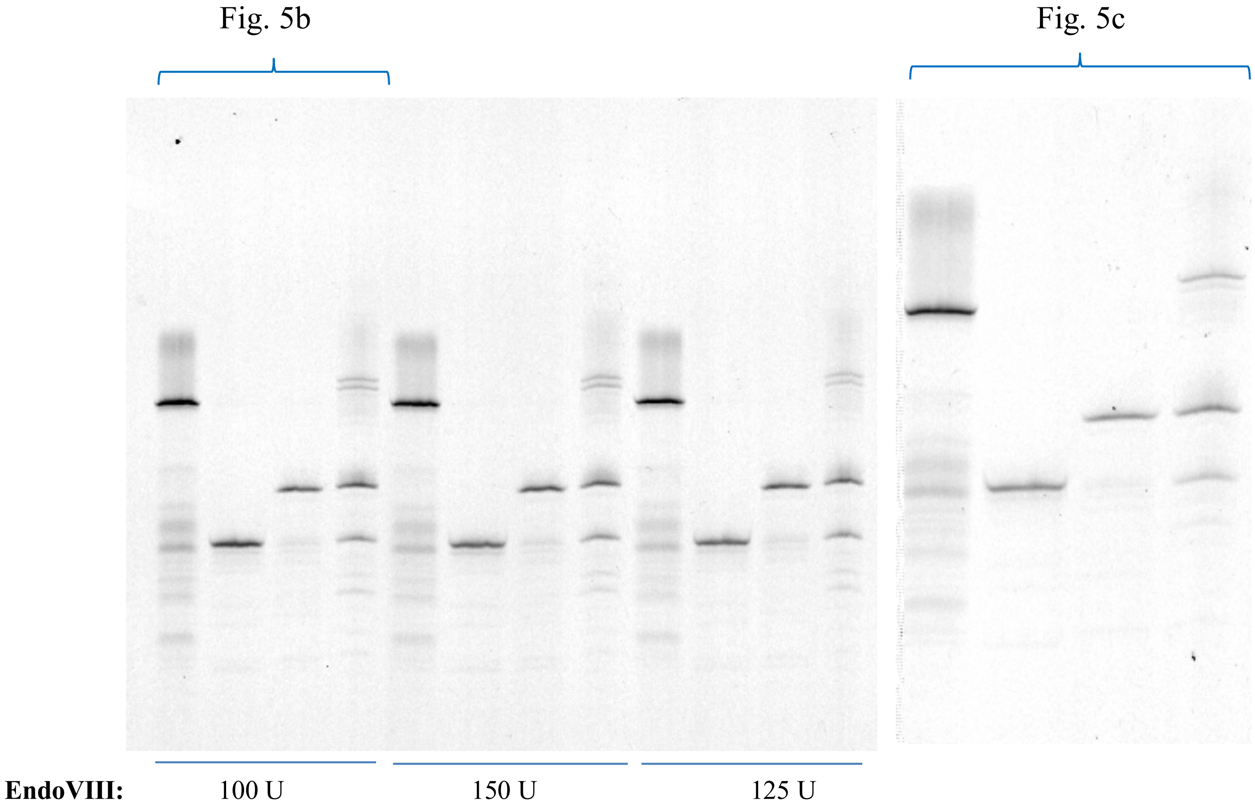

Supplementary Figure S6. *Full image of gels shown in Figure 5 of the main text, showing results of the ligation protocol with EndoVIII (left, including additional titrations) and with phenol (right)*

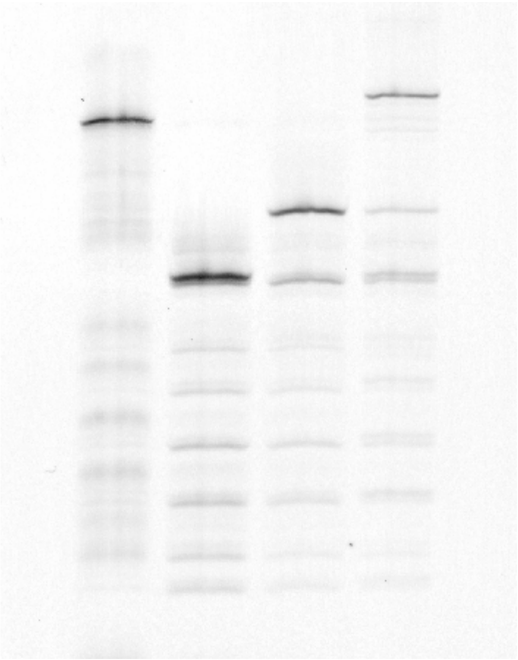

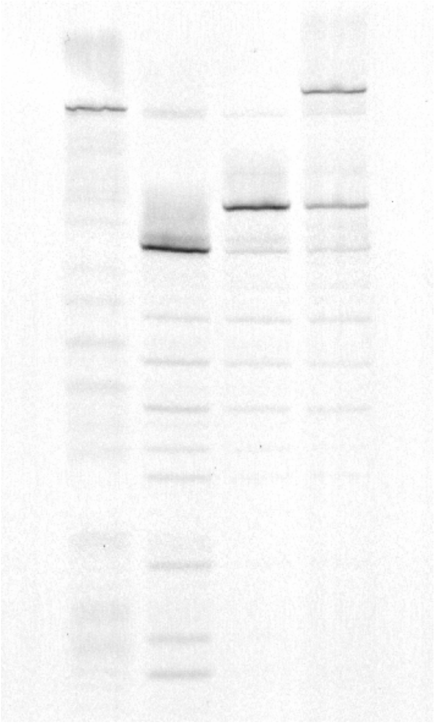

Supplementary Figure S7. *Full image of gels shown in Supplementary Figures 4 (left) and 5 (right)*

Supplementary Methods

Data in Supplementary Figure S2:

Chemoenzymatic generation of DNA substrates 54 nt template DNA was purchased from Integrated DNA Technologies (Coralville, IA) having the sequence 3'-TAG AGT GAT GTT TGG TAG ATG TTA CCG GCT ACT AAC CCA TAC AAC TAT CTT CAT-5'. To produce a duplex construct with a single 5mC, 5hmC, or 5fC, a 27 nt primer strand featuring a 5'-FAM was first purchased (Integrated DNA Technologies) with sequence homology to the 3' end of the template strand. Upon annealing, the unpaired bases of the template contained only one G (at base position 28). Consequently, primer extension was be carried out with canonical dATP, dGTP, dTTP, and either 5-methyl-dCTP, 5-hydroxymethyl-dCTP, or 5-formyl-dCTP (Trilink BioTechnologies, San Diego, CA) using Klenow DNA polymerase (New England Biolabs, Ipswitch, MA) to result in the insertion of a single modified base in the final construct. Excess dNTPs were removed using a commercial kit (Oligo DNA Clean & Concentrator kit, Zymo Research, Irvine, CA).

TET reactions 500 nM of DNA substrates were then reacted with 3.5 μM TET2-CS in 50 mM HEPES (pH 6.5), 100 mM NaCl, 1 mM α-ketoglutarate, and 2 mM L-ascorbic acid using 75 μM $Fe(NH_4)_2(SO_4)_2$ as an initiator of the reaction in a total volume of 10 μL. After 30 min at 37 ºC, the reactions were cleaned up by Oligo DNA Clean & Concentrator kit (Zymo) and eluted into 10 μL of water. 2.5 μL of the resulting sample was then incubated for 4 hrs with TDG (1 μM) in 10 mM Tris-Cl (pH 7.5) and 100 mM NaCl. The reactions were terminated and abasic sites finally cleaved with 166 mm NaOH at 85 ºC for 5 min. The samples were mixed with formamide loading dye and run on a 12% denaturing PAGE.

Data from Supplementary Figure S4:

OxoG labeling A custom 40 nt oligonucleotide (Integrated DNA Technologies) with a 5' FAM (sequence: TCA CGA CTA GTG TTA ACA TGT GCA CCT **G^o**CA GAA TGA GAA T, where **G^o** is oxoG) was annealed to a complementary sequence by mixing both at an equimolar ratio, incubating in deionized water at 95°C for 10 minutes, and cooling to room temperature over 1 hour. To excise oxoG, a 30 μL aliquot was prepared containing 50 pmol of duplex, 16 U FPG (New England Biolabs), 20 U EndoIV (New England Biolabs), 3 μg bovine serum albumin (New England Biolabs), and incubated in 1X NEB2 buffer (New England Biolabs) at 37°C for 1 hr. Next, 0.75 nmol of biotinylated dGTP (Perkin Elmer, Waltham, MA) and 0.06 U T4(exo-) (Lucigen, Middleton, WI) were added to a final volume of 40 μL in 1X NEB2 buffer and the mixture was incubated at 37°C for 30 minutes. Finally, the mixture was subjected to the QIAquick PCR purification kit (Qiagen, Valencia, CA) to remove proteins and excess nucleotides. 40 pmol of the resulting DNA was incubated with 400 U of T4 DNA Ligase (New England Biolabs) in T4 DNA Ligase buffer (50 mM tris-HCl 10 mM, $MgCl_2$, 1 mM ATP, 10 mM DTT, pH 7.5) overnight at room temperature. The DNA was then purified by QIAquick PCR purification kit and eluted in deionized water.

Data from Supplementary Figure S5:

Uracil labeling and ligation A custom 40 nt oligonucleotide (Integrated DNA Technologies) with a 5' FAM (sequence: TCA CGA CTA GTG TTA ACA TGT GCA CCT GCA GAA **U**GA GAA T) was annealed to a complementary sequence by mixing both at an equimolar ratio, incubating in deionized water at 95°C for 10 minutes, and cooling to room temperature over 1 hour. To excise uracil, a 30 μL aliquot was prepared containing 100 pmol of duplex DNA, 20 U *E. coli* UDG (New

England Biolabs), 40 U EndoIV, 3 μg bovine serum albumin, and incubated in 1X NEB2 buffer at 37°C for 1 hr. Next, 1.5 nmol of biotinylated dUTP (Perkin Elmer), and 0.12 U T4(exo-) were added to a final volume of 40 μL in 1X NEB2 buffer and the mixture was incubated at 37°C for 30 minutes. Finally, the mixture was processed with a QIAquick PCR purification kit to remove proteins and excess nucleotides. 40 pmol of the resulting DNA was incubated with 400 U of T4 DNA Ligase in T4 DNA Ligase buffer (50 mM tris-HCl 10 mM, $MgCl_2$, 1 mM ATP, 10 mM DTT, pH 7.5) overnight at room temperature. The DNA was then purified by QIAquick PCR purification kit and eluted in deionized water.
